# Stem Cell Transplantation for Parkinson’s Disease: Current Challenges and Perspectives

**DOI:** 10.14336/AD.2022.0312

**Published:** 2022-12-01

**Authors:** Xinlin Zeng, Hua Qin

**Affiliations:** ^1^Nankai University, College of Life Sciences, Tianjin 300071, China; ^2^Research Center for Tissue Repair and Regeneration affiliated with the Medical Innovation Research Division and 4^th^ Medical Center, PLA General Hospital and PLA Medical College, Beijing 100853, China.

**Keywords:** stem cell transplantation, Parkinson’s disease;, dopaminergic neuron, neuroinflammation, neurogenesis

## Abstract

Parkinson’s disease is the second most common form of neurodegeneration, and it poses a major threat to the quality of life of older adults. Stem cell transplantation, which has attracted widespread attention from researchers, is a new treatment that is demonstrating excellent potential for treating Parkinson’s disease. This paper introduces the advantages, disadvantages, and current research on the progress of using stem cells for Parkinson’s disease; briefly describes the strategies for controlling the differentiation of stem cells into dopaminergic neurons in vitro; highlights how transplanted cells improve the loss of dopaminergic neurons by interacting with the inflammatory microenvironment in the brain; and proposes that future stem cell research focus on finely regulating the signal pathways that influence the directed differentiation of dopaminergic neurons to maintain an appropriate balance between the modulatory factors that affect the inflammatory microenvironment and clarify the interaction between neurons and neuroglia.

## Introduction: Pathology and current treatment strategies for Parkinson’s disease

Parkinson’s disease (PD) is one of the most prevalent neurodegenerative disorders among the population aged over 60. The most notable features of PD are the progressive degeneration of dopaminergic (DA) neurons in the substantia nigra pars compacta, which is a region in the ventral midbrain (VM), and the aggregation of α-synuclein (α-syn) into Lewy bodies (LBs) [1]. Because of the loss of dopamine storage, patients with PD exhibit severe symptoms that are associated with decreased motor function, including resting tremors, bradykinesia, rigidity, postural instability, and the gradual impairment of autonomic, cognitive, and mood functions [2].

The cause of PD remains unclear, but the prevailing view is that it is a multifactorial disease in which genetic predisposition, aging, neuroinflammation, and oxidative stress play key roles [3]. Extensive evidence indicates the presence of increased oxidative stress (including the increased oxidation of lipids, DNA, and proteins) in patients with PD. Dopaminergic neurons oxidize dopamine through monoamine oxidase, causing the production of superoxide and hydrogen peroxide. This means that dopaminergic neurons are in a perpetual state of oxidative stress, which may lead to reduced levels of endogenous antioxidants [4]. The involvement of mitochondrial function in PD also supports this hypothesis because the neurotoxin 1-methyl-4-phenyl-1,2,3,6-tetrahydropyridine (MPTP), which can inhibit mitochondrial respiratory complex I, can lead to PD-like pathologies in humans [5]. In addition, the discovery of epigenetic mechanisms related to brain development and neuronal differentiation has given epigenetic dysregulation a key role in age-related neurodegenerative diseases [6]. For example, the c-Abl-mediated phosphorylation and inhibition of parkin ligase function can result in the accumulation of parkin interacting substrate, which activates p53-dependent cell death pathways in dopaminergic degeneration [7]. Upregulated lncRNA HOXA-AS2 interacts with polycomb repressive complex 2 (PRC2) and modulates the histone methylation of the promoter of PGC-1 alpha to promote microglial polarization, which is a crucial pathology in the neuroinflammation of PD [8].

To date, PD lacks highly effective treatments. Commonly prescribed drugs act on malfunctioning dopaminergic neural networks by acting as levodopa or dopaminergic receptor agonists to reduce dyskinesia. This treatment is highly effective in the early-stage PD but causes severe side effects, such as movement complications (fluctuating symptoms and dyskinesia), hallucinations, and various psychiatric symptoms. Deep brain stimulation and the subcutaneous injection of apomorphine can be used as alternative therapies when motor complications cannot be controlled by drugs during late-stage PD. However, the limitations of the drugs include their limited effects on nonmotor symptoms and a high incidence of related adverse events, infections, cerebral hematoma, and psychological and behavioral abnormalities [9].

Stem cells are cells that can produce progeny and differentiate into multiple cell lineages. Because the degeneration of dopaminergic neurons in PD is largely irreversible, establishing multifarious stem cell lines, promoting their directional differentiation into dopaminergic neurons, and promoting the integration of stem cells into the central nervous system (CNS) to improve the clinical effects of stem cell transplantation appear to be promising strategies for treating PD.

## Selection of appropriate stem cell sources: Cell replacement therapy candidates for generating sufficient neural lineage-specific cell lines

At present, various types of stem cells are used to meet human needs, and the most widely used stem cell sources for DA neuron replacement are human embryonic stem cells (hESCs), human neural stem/progenitor cells (hNSCs/hNPCs), human mesenchymal stem cells (hMSCs), human fetal ventral midbrain cells, and human-induced pluripotent stem cells. In the present article, we introduce studies that have explored the use of embryonic stem cells (ESCs), neural stem cells (NSCs), mesenchymal stem cells (MSCs), and pluripotent stem cells (iPSCs) in PD therapy and compare their advantages and disadvantages (Table 1).

**Table 1:** The advantages and disadvantages of different stem cell types.

Stem cell types	Advantages	Disadvantages
ESCs	1.stable development potential to form all three embryonic germ layers 2.prolonged undifferentiated proliferation 3.survive transplantation and rescue functional deficits	1.limited tissue availability 2.ethical concern 3.possibility of teratoma generation 4.immune rejection
NSCs	1.ability to expand and differentiate into various neurons and glia 2.reduced tumorigenic potential and immunological rejections	1.limited tissue availability 2.ethical concern 3.limited lineage differentiation
MSCs	1.easily accessible source tissue 2.not burdened with ethical issues	1.modest functional recovery in human 2.biodistribution related toxicity
iPSCs	1.histocompatibility 2.survive transplantation and rescue functional deficits 3.easily accessible source tissue 4.minimal immunorejection and ethical issues	1.genetic and epigenetic alterations 2.low reprogramming efficiency 3.tumorigenic hazards

ESCs, Embryonic stem cells;NSCs, Neural stem cells; MSCs, Mesenchymal stem cells; iPSCs: Induced pluripotent stem cells.

### Embryonic Stem Cells: Stable development potential for yielding a limitless number of homogenous DA progenitors

ESCs are derived from the inner mass of a developing blastocyst; they can self-renew and differentiate into cells of three primary germ layers (i.e., ectoderm, mesoderm, and endoderm) under specific circumstances and have been used in transplantation experiments involving PD animal models and patients with PD. Human ESC-derived dopaminergic neurons that were transplanted to rat striatum lesioned with the neurotoxin 6-hydroxydopamine have successfully survived and cooperated with other newly differentiated cells, leading to locomotive function recovery at 5 months [10]. However, ESC transplantation is affected by various problems. Lack of access to source tissue and ethical concerns are key factors that limit the development of ESC-based strategies. In addition, grafted ESCs may cross the blood-brain barrier and functionally integrate into ectopic brain regions and secrete active factors that adversely affect the CNS. In addition, hESCs and their derivatives may induce immune rejection after they are grafted into a recipient’s brain [11].

### NSCs: A remedy that allows endogenous NSC pools to obtain various neurons and glia

NSCs are the least committed adult stem cells of the nervous system, and they have self-renewal and multipotency abilities to generate all neuroectodermal lineages (including neurons, astrocytes, and oligodendrocytes) depending on the regional and developmental stage. In mammals, NSC-based neurogenesis occurs in brain sites called niches, which include the subgranular zone of the hippocampal dentate gyrus, the subventricular zone (SVZ) of lateral ventricles, and the external germinal layer of the cerebellum [12]. A substantial body of evidence indicates that the NSCs in the SVZ can migrate to the olfactory bulb to become new functional neurons in rodents, but this process has not yet been observed in adult humans. Human experimental data indicate that hippocampal neurogenesis diminishes considerably over time. However, when NPCs are used for stem cell transplantation, they have clear advantages over ESCs in reducing tumorigenic potential and immunological rejection; thus, NSC transplantation is a potential strategy for PD therapies. In one study, the NSCs taken from the VM of rats grew and expanded in a basic fibroblast growth factor (bFGF)-embedded system and then differentiated into numerous tyrosine hydroxylase positive (TH+) neurons, leading to functional recovery when they were grafted into rat PD models [13]. However, obtaining a sufficient amount of adult human CNS tissue for the preparation of adult NSCs is a difficult task.

### MSCs: A readily accessible cell type that warrants further research

MSCs are a type of nonhematopoietic, multipotent subtype cell line, and they originate mainly from the stromal structures of the bone marrow (excluding the adipose tissue, umbilical cord, dermis, and peripheral blood). MSCs have the transdifferentiation potential to form the neurogenic potential for transdifferentiating into neurospheres under specific circumstances [14]. Because they are obtainable from multiple sources, MSC-based treatments have been widely used in various PD animal models. For example, bone marrow stromal cells can be induced to differentiate into neuron- and glia-like cells by using brain-derived neurotrophic factors (BDNFs), bFGF, epidermal growth factors (EGFs), and glial-cell-line-derived neurotrophic factors (GDNFs), all of which can be transplanted into the CNS to promote motor recovery [15]. Compared with ESCs and NSCs, MSCs are unaffected by ethical and immunological rejection problems. However, in clinical trials involving MSC transplantation, the proliferation of MSCs was nonsignificant, which raised questions about the efficacy of MSC transplantation-based treatments [16]. This may be because MSCs promote regeneration mainly through the secretion of biologically active factors. Thus, the use of extracellular vesicles (EVs) derived from MSCs, which have microRNAs (miRNAs) and proteins that repair or rejuvenate the brain, is a novel alternative to conventional MSC-based therapies [17].

### Induced NCSs: Potential for autologous cell transplantation

iPSCs are ESC-like pluripotent cells; they are obtained from reprogrammed embryonic and adult mouse fibroblasts by retrovirally introducing Oct3/4, Sox2, Klf4, and c-Myc (OSKM) gene transcription factors [18]. For PD research, iPSC technology can promote the generation of midbrain DA neurons, which may play a significant role in basic research on the detailed molecular mechanisms of PD and stem cell therapy that involve the use of autologous donor cells. In addition, the utilization of iPSCs from patients with PD provides an opportunity for autologous transplantation, which considerably reduces graft rejection. For the aforementioned reasons, iPSCs are applied frequently in practical animal model tests. Studies have shown that transplanted human iPSC-derived NPCs pretreated with multiple factors can generate numerous functional DA neurons in the monkey brain [19]. However, a concern is that the employment of integrating viruses (especially the introduction of integrated oncogenes such as c-Myc and Klf4) is likely to change the genome of host cells, which poses a considerable threat to human health and safety. Therefore, new reprogramming methods (e.g., methods involving the use of miRNAs, small-molecule compounds, nonintegrating vectors, and cell membrane-permeable proteins) have been applied [20]. Among them, the use of small-molecule compounds is highly promising for clinical application because it provides several unique advantages, such as structural versatility, controllability in a time- and concentration-dependent manner, and a low cost for mass production. For example, the combination of the four small molecules VPA, tranylcypromine, CHIR99021, and 616452 can replace Sox2, Klf4, and c-Myc in their role in inducing reprogramming with just the single transcription factor Oct4 [21].

## Control of stem cell differentiation: More standard protocols, delicate regulation, and homogeneous differentiation

Stem cells are used to replace the dopamine-producing neurons in the substantia nigra that are progressively lost during PD progression. However, because differentiation in vivo is difficult to control accurately and the proliferation of undifferentiated stem cells is extremely high, direct transplantation often carries a high risk of tumorigenicity, and thus, the optimal method for using stem cells is to transplant them after they have been directed to differentiate into specific types of mature cells in vitro [22]. Stem cell therapy should fulfill several key criteria to become an effective and competitive therapy. These criteria include the ability to produce sufficient cells, especially substantia nigra dopaminergic neurons that can integrate into the host brain after transplantation and form synapses with other neurons. Furthermore, the transplanted cells should not exhibit or should only exhibit very low levels of proliferation to prevent the formation of tumors at the site of grafting. Here, we introduce several prevalent strategies for controlling stem cell differentiation.

### Regulation of signaling pathways: Widely used differentiation induction strategies based on key determinants of dopaminergic cell fate

**Figure 1.**
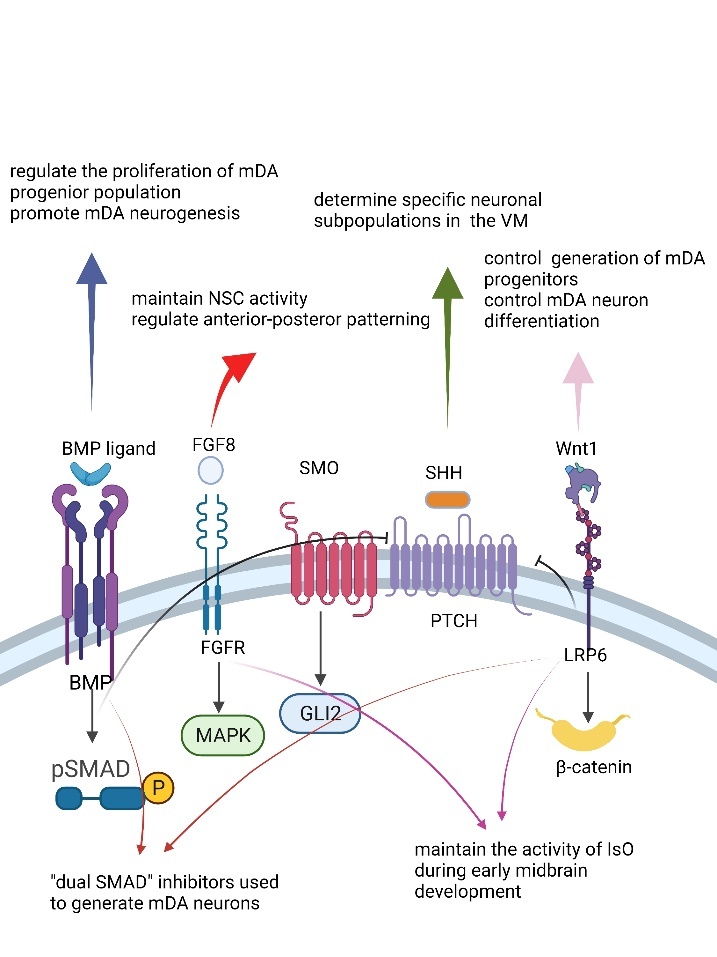
The function and crosstalk of important signaling pathways in mDA neuron development. Schematic and simplified depiction of the components, function and crosstalk of the main signaling pathways involved in the generation and maintenance of mDA neurons in the mammalian embryo, BMP, FGF, SHH and WNT/β-catenin (from left to right).

During embryonic development, developmental patterns and cell fate-specific differentiation are regulated by locally secreted morphogens and growth factors. The time and space dimensions of developmental patterns are controlled by the gradient of the concentration of morphogens distributed along the anterior-posterior axis and the dorsal-ventral axis. Fibroblast growth factor, WNT, and retinoic acid influence the anterior-posterior pattern, and the classical WNT/β-catenin signaling pathway plays a key role. The components that affect the dorsal-ventral pattern include WNT, bone morphogenetic proteins, and sonic hedgehog (SHH) signaling pathway. The concentration gradient of morphogens along the axis of the anterior-posterior pattern and dorsal-ventral pattern defines the transcriptional code and the identity of nerve precursor cells in specific regions. A slight change in the concentration of morphogens can cause a change in the fate of a cell. Therefore, most in vitro differentiation induction protocols have focused on regulating the aforementioned signaling pathways. The methods for regulating signal pathways can be mainly divided into two categories; the first is genetic manipulation involving the activation or inhibition of specific fate-determining transcription factors, and the second is the use of chemically defined human additives to specifically activate or inhibit key pathways (Fig. 1).

bFGF promotes the differentiation of multiple types of stem cells, such as NSCs, ESCs, and MSCs [23]. In a study, bFGF was demonstrated to protect dopamine neurons that were isolated from the substantia nigra and ventral tegmental area of the rat midbrain when it was exposed to MPP+(1-Methyl-4-phenylpyridinium iodide) [24]; the study revealed that bFGF can promote the proliferation of bone marrow MSCs in vitro, which can differentiate into dopaminergic neurons. In addition, the cotransplantation of bFGF and hBMSCs(Human bone marrow-derived mesenchymal stem cells) can improve motor dysfunction in PD rats in vivo [25].

The SHH pathway is crucial for establishing protocols that generate numerous midbrain dopaminergic (mDA) neurons for human PSCs. Researchers who have attempted to generate mDA neurons from PSCs for PD cell replacement strategies have encountered difficulties relating to the phenomenon of generated neurons not displaying all the principal characteristics of authentic mDA neurons (e.g., FOXA2 expression) and exhibiting poor survival after transplantation. Subsequently, the establishment of floor plate (FP) cells as a cell lineage that is different from BP neuronal progenitors during early neural development led to a breakthrough in the generation of authentic mDA neurons in vitro. Scientists discovered that human ESCs can be directly induced into FP cells that have anterior characteristics through the addition of high doses of SHH at early stages of neural differentiation. The addition of retinoic acid, FGF8, or WNT1 together with SHH can cause a posterior shift of their fate. In particular, WNT1 resulted in the generation of FP cells with midbrain phenotypes [26]. These findings were utilized to generate mDA neurons from human PSCs; specifically, SHH signaling and FGF8 signaling are activated from days 1-7 of neural differentiation, and WNT signaling is activated from day 3 onward. After 11 days, this protocol yields midbrain FP cells, and after 25 days, it yields neurons with characteristic expression types that are similar to those of mDA neurons [27].

Although two morphogens have been verified to be essential for mDA neuron induction, scientists have also discovered that WNT1 is required to induce ectopic mDA neurons from VM NSCs [28]. Experiments have revealed that the role of WNT signaling in the differentiation of mDA neurons is ligand- and dose-dependent. The addition of WNT1 or WNT/β-catenin agonists (e.g., the GSK3β inhibitor CHIR/CT99021) promotes and inhibits the generation of TH+ mDA neurons at low and high dosages, respectively [29]. Researchers have noted that only the early application of intermediate concentrations of CHIR/CT99021 can produce mDA neurons that can functionally rescue movement deficits in rodent PD models [30].

In addition to the aforementioned signaling molecules mentioned, other transcription factors (e.g., Nurr1, Lmx1a, and Msx1) also participate in the process of DA cell fate specification, and thus, they are promising tools for generating DA neurons from stem cells [31, 32]. In conclusion, the elaborate genetic regulation of signaling molecules and key factor exposure in culture systems under tight temporal and spatial control can synergistically enable the generation of DA neurons from stem cells.

### Addition of growth factors, cytokines, neurotrophic factors: Combined regulation of exposure to various key factors

Adding various growth factors, cytokines, extracellular matrix proteins, neurotrophic factors, and neuroregulatory molecules to induce the differentiation of stem cells into dopaminergic neurons is an efficient method that is widely used. BDNFs, GDNF, and insulin-like growth factor 1 are the most frequently studied neurotrophic factors of the CNS. Research has indicated that these factors play key roles in the growth and stabilization of dendritic spines, synaptic plasticity, and survival of neurons and glia; consequently, they are closely related to neurodegeneration. For the proliferation and differentiation of NSCs, Studer reported that the NSCs from the VN of rats grew and expanded in a bFGF-embedded system and then differentiated to generate numerous TH+ neurons, which could induce functional recovery upon being transplanted into rat PD models [13].

The addition and combination of various soluble factors, neurotrophic factors, and cytokines enhances the production of DA neurons. For example, compared with the use of FGF2 alone, a combination of BDNF and FGF2 allows for the efficient generation of functional dopaminergic neurons from human MSCs [33]. The inhibition of HPHs can stabilize HIF-1; this mechanism was demonstrated to enhance the dopaminergic differentiation of stem cells and protect dopaminergic neurons against neurotoxins; thus, it is a promising strategy for cell replacement therapies [34].

### Regulation of miRNAs: Promising targets for manipulating stem cell fate and controlling stem cell differentiation

miRNAs are conserved small (~22 nt) noncoding RNAs that are involved in the pathogenesis of numerous diseases, apoptosis, aging, cell fate decisions, cell cycle regulation, and multiple signaling pathways. miRNAs can specifically recognize and regulate the expression of target mRNAs through sequence complementarity within the untranslated region of mRNA 3′. Moreover, numerous steps of neurogenesis, from NSC self-renewal and fate determination to neuronal maturation, are regulated by miRNAs. miRNAs play a key role by targeting downstream genes, from transcription factors to epigenetic regulators, in a dynamic and context-dependent manner [35]. In a study, the profiles of miRNA expression in iPSCs and NPCs were compared by conducting a microarray-based analysis [36] to clarify the role of miRNA in controlling stem cell differentiation.

Crucially, the stem cell derivation, maintenance, and neuronal differentiation of NSCs are tightly regulated by miRNAs. For instance, the overexpression of miR-124 in HeLa cells can change their transcriptome such that it becomes highly similar to that of a neuronal expression profile; this simultaneously improves the expression levels of neuronal genes and represses the expression of nonneuronal genes [37]. Furthermore, researchers have demonstrated that neurons can be produced from human fibroblasts with the use of miRNAs [38]. Therefore, miRNAs are highly promising therapeutic agents or targets for manipulating the aforementioned cells in the desired manner.

Moreover, miRNAs are closely related to dopaminergic neuron differentiation. The WNT1-cre-mediated conditional knockout of dicer results in the occurrence of numerous developmental brain defects in mice, including a lack of expression of miR-124, miR-9, and miR-218 that causes the severe developmental impairment of dopaminergic neurons. In addition, the miRNA expression profiles in patients with PD exhibit a substantial reduction in miR-133b levels. In PD mice, the expression of miR-133b is significantly reduced. In fact, a feedback loop exists in dopaminergic neurons where Pitx3 specifically induces the transcription of miR-133b, which in turn downregulates Pitx3 expression. The aforementioned findings suggest that the maturation and maintenance of dopaminergic neurons are controlled by miR-133b [39]. In addition, the ectopic expression of miR-132 in embryonic stem cells may reduce the number of differentiated TH+ neurons by downregulating Nurr1. MiR-7 negatively regulates the proliferation of DA progenitor cells by inhibiting the Wnt-β-catenin signaling pathway in zebrafish embryos and positively regulating the SHH signaling pathway to control the balance between oligodendrocytes and DA neurons [40].

## Challenges and prospects

Recently, numerous cell replacement therapy (CRT) studies of PD have focused on replacing degenerated dopaminergic neurons by inducing DA progenitor cells from stem cells in vitro before transplantation. At the time of writing, numerous differentiation protocols are based on the key method of inhibiting Smads, adding high levels of SHH, and activating WNT1 to generate FP cells and midbrain dopaminergic neurons. Researchers are exploring differentiation-induction strategies that exhibit increased refinement and effectiveness; such strategies include the application of all-trans-retinoic acid, SHH, FGF, proepidermal growth factor, bone morphogenetic protein, and neurotrophin to activate critical transcription factors such as Nurr1, Lmx1a, Pitx3, PAX4, GATA, and coculturing feeder cells.

Although stem cell replacement therapy for PD is starting to enter its clinical phase, numerous problems must still be addressed and solved. Under normal conditions, the NSCs in the ventricular zone of the midbrain migrate radially to the median zone, where they develop into neural blastocysts; they then continue to migrate to the marginal zone, where they develop into A9 dopaminergic neurons. They then migrate tangentially to the pars compacta, where the cell types are identical (i.e., exhibiting uniformity). This process requires precise temporal and spatial control of various morphogens to complete. However, an in vitro differentiation protocol for achieving such homogeneous differentiation has yet to be developed. The cells that are obtained are usually a mixture of dopaminergic neurons and other atypical cell types. This heterogeneity can lead to tumor formation and dyskinesia in the host after transplantation, which leads to potential and unpredictable safety risks for patients. Therefore, in addition to midbrain dopaminergic neurons, the identification of other cell types, the cause of their formation, and the methods for eliminating them are key questions to address in the future.

Notably, several studies have revealed that transplantation with undifferentiated NSCs also leads to considerable behavioral recovery in both rodent and primate PD models. In contrast to the induction of differentiation in vitro before grafting, NSCs can not only generate a small population of dopaminergic neurons in vivo but also provide multiple benefits, including neurotrophic support, brain vascularization regulation, and immunomodulation. Preclinical studies on rodent and nonhuman primate models of PD have demonstrated that successfully engrafted hpNSCs survive for a long period of time, increasing the dopamine level and fiber innervation in the brain and promoting behavioral recovery without causing serious adverse events (e.g., dyskinesia, tumors, and ectopic tissue formation) [41, 42]. Although clinical trials have been conducted, concerns still exist regarding the possibility of NSCs differentiating into other types of neural cells, which may cause dyskinesias and dangerous mutations during their division. Researchers are still debating whether the implantation of a pure population of DA progenitors/neurons or undifferentiated NSCs is more favorable. Thus, further research should be performed to evaluate the ability of NSCs to generate specific cell types and cause immunological rejection and biodistribution-related toxicity after NSC transplantation.

For CRT, future studies should explore methods that enable the safe, accurate, and reproducible generation of dopaminergic neurons, the sources of cells that can be used (e.g., ESCs, NSCs, and iPSCs), methods for grafting the aforementioned cells (e.g., use of pure dopaminergic neurons or supporting midbrain glial cells), the developmental stages (e.g., undifferentiated stem cells, DA progenitor cells, or DA neurons) for which the aforementioned methods are applicable, and the sites where the aforementioned cells should be grafted to achieve a more favorable response (e.g., substantia nigra or putamen).

## Enhancement of host brain microenvironment: Rate-limiting process for transplanted cells that survive and integrate into a host’s neural circuits

In neuropharmacological mechanisms, the primary roles of stem cells are neuroprotection and neurogenesis. For PD, neurogenesis refers to the proliferation of endogenous NSCs, which yield functional dopaminergic neurons and form new synapses with endogenous neurons [43]. However, the clinical test results obtained through current NPC transplantation methods are generally regarded to be inadequate for meeting the needs of patients. Research indicates that the number of stem cells that survive transplantation and integration into brain tissue in vivo is usually low. Thus, most of the therapeutic benefits of NSC transplantation may likely be due to not only the replacement of cells but also the beneficial bystander effects of NSCs on the microenvironment.

### Modulation of immune system: Beneficial bystander effects that regulate neuroinflammation

The neuroprotective ability mainly results from undifferentiated stem cells releasing various types of neuroprotective molecules at the site of tissue damage, thereby containing immunomodulatory substances and neurotrophic growth factors in accordance with temporal and spatial environmental needs. In neurodegenerative diseases such as PD, neuroinflammation is a crucial phenomenon that influences the survival of cells; the inflammatory environment also influences an NSC niche, thereby affecting stem cell survival, self-renewal, migration, and differentiation [44].

The role of inflammation varies depending on the grafting environment or transplantation stage; it either amplifies or suppresses the activity of stem cells. The immune response following stem cell transplantation is a highly debated topic. Usually, anti-inflammatory cytokines such as interleukin (IL)-10 and TGF-β are perceived to have a positive effect on neurogenesis, whereas proinflammatory cytokines such as IL-1, IL-6, and TNF-α are perceived to hamper neurogenesis. For example, NPCs can help host cells to escape inflammation-induced cell death by regulating the levels of key inflammatory molecules such as TNF-α and IL-6 [45]. hNSC transplantation has been demonstrated to promote the dedifferentiation of local astrocytes, inhibition of microglia and proinflammatory cytokines, and generation of neurotrophic factors; therefore, it can provide neuroprotective benefits by regulating a host niche [46].

### Stem cell-sourced secretome: Excellent drug delivery vehicles

Because grafted cells may trigger allogeneic immune responses and aggravate ongoing inflammation, which then have severe effects on patient safety, scientists are exploring other effective but safer therapies for patients with PD. Thus, the new concept of secretome was developed. The regenerative potential of the secretome can be explained by the bystander effect hypothesis, which refers to the ability of stem cells to release useful substances to improve neural regeneration and restore motor function [16]. Therefore, the use of stem cell-derived secretomes to treat neurodegenerative diseases may be a promising option.

An MSC-sourced secretome combines MSC-derived bioactive factors such as nucleic acids, lipid soluble proteins, and EVs. A study demonstrated that adding hMSC-derived EVs to cultures can protect NSC-derived dopaminergic neurons from 6-hydroxydopamine (6-OHDA). Another study reported that intranasally administered MSC-EVs had significant curative effects in a 6-OHDA model of rat PD [47]; thus, it demonstrated that the EVs from MSCs can reduce parkinsonian symptoms in a rat model.

EV-based drug delivery methods have several advantages. For instance, EVs efficiently carry cargo-containing proteins and miRNAs from parental cells to recipient cells. The immune response can be reduced if it is derived from the same species, and EVs can be incorporated into target cells. Therapeutic EVs, such as those derived from MSCs and NSCs, can also be modified through molecular engineering techniques to carry protein ligands for targeted delivery to specific cells in a body [48]. For example, scientists have developed EVs that are loaded with catalase, which is an effective antioxidant. In PD models, catalase was demonstrated to improve neuroinflammation and neuronal survival when inflamed neurons absorb catalase exosomes. MSCs can also host beneficial miRNAs, thereby reducing neuroinflammation and promoting neurogenesis in patients with PD [49]. The downregulation of miR-133b in PD models can be rescued to normal levels through MSC-EVs, which promote neurite growth [50]. The aforementioned results all demonstrate that the transfer of MSC exosomes is effective in treating animal PD models.

## Challenges and Prospective

The survival of transplanted cells and their integration into a host’s neuronal circuits are rate-limiting processes that influence the success of CRT. Current stem cell transplantation therapy has several key problems. One problem is the immune rejection of grafted cells. Although the brain is immunologically privileged (i.e., the ability to tolerate the introduction of histoincompatible transplants without generating a full immune rejection response), this property is not always present. Therefore, even if the grafted cells used on a patient are derived from that patient (e.g., autologous iPSCs), they are not completely devoid of rejection risk. To solve this problem, human leukocyte antigen (HLA) matching before grafting and immunosuppressive drug treatment are usually required. However, the appropriate type, dose, and duration of immunosuppressive drug treatment have yet to be established. Most clinical programs adopt organ transplant protocols and will gradually reduce or halt immunosuppression several months or years after surgery. Of note, CRISPR/Cas9-mediated genome editing system can be used to knock out relevant HLAs and costimulatory molecules expressed on cells to reduce the occurrence of immune rejection responses [20].

The other challenge regarding the application of cell-based replacement strategies for PD is that grafted cells may become diseased in a diseased brain microenvironment. First, for autologous iPSC-derived dopaminergic neurons, the grafted cells that are used on a patient may develop PD pathology because they are derived from that patient rather than from healthy individuals, and heritability can influence overall PD risk. Second, even if allogeneic iPSC-derived dopaminergic neurons are used, normal neurons are still embedded in diseased brain tissue, where they may be exposed to various deleterious risk factors, such as neuroinflammation and toxic misfolded proteins. In this situation, the transplanted cells may gain a similar PD pathology over time, leading to the loss of their function and eventually their death.

The aforementioned findings indicate that how to improve the brain microenvironment of a host is a crucial topic to address in the future. The prospective methods for improving a brain microenvironment include maintaining the balance between proinflammatory and anti-inflammatory factors, applying the neuroprotective roles of neurotrophin, and activating neuroprotective signaling pathways (e.g., Nrf2-ARE, and cografting astrocytes), which can provide physiological functions to support neuronal cell survival, neuronal function, and brain homeostasis.

## Discussion

Various types of stem cells are currently used for PD therapy. In the present article, we discussed ESCs, NSCs, MSCs, and iPSCs, all of which have been demonstrated in numerous in vitro and in vivo experiments to be effective for PD models. However, each of them has its own set of major problems that should be solved to obtain more favorable outcomes for clinical applications. Compared with ESCs and NSCs, MSCs probably have greater therapeutic potential for PD because they are not prone to tumor formation and immunological rejection after transplantation. They are unaffected by ethical problems, are more accessible, and are not prone to immunological rejection because of their lack of major histocompatibility complex III. However, more investigations should be conducted to examine the biodistribution-related toxicity, pale reprogramming efficiency, and modest clinical improvement of MSCs in accordance with existing MSC transplantation practices. iPSCs can be obtained directly from the cells of patients, and thus, iPSC use reduces the risk of transmissible infections and immunological rejections and allows for the implementation of personalized medicine. However, the autologous transplantation of patient-specific iPSCs may also expose the cells of a patient to the risk of existing pathology and make them susceptible to an inflammatory microenvironment.

Numerous viable strategies are currently applied to induce dopaminergic neuron differentiation in vitro for transplantation. However, key challenges and limitations remain that must be overcome before the aforementioned strategies can be applied in clinical trials. A major challenge is the considerable differences among the protocols and cells that are used to generate mDA neurons, especially with respect to the complexity of stem cell therapies and the numerous inherent differences among various stem cell lines. Thus, the construction of optimal and standardized mDA neuron-inducing protocols for clinical translation is highly challenging but meaningful. In addition, heterogeneity is also a critical problem. The differentiated cell population that is obtained through current methods is a mixture of dopaminergic neurons and other cell types. This heterogeneity can cause tumor formation and dyskinesia in a host after transplantation, which introduces potential and unpredictable safety risks for patients. In most current differentiation protocols, the efficiency for obtaining SNc/A9 dopaminergic neurons is generally between 40% and 50%, although it can reach 75%. In summary, heterogeneity is present in the dopaminergic neural differentiation products of stem cells, and further optimization is required. The first optimization method involves the seeking of stem cell markers that can be specifically differentiated into A9 dopaminergic neurons and subsequent screening of specific stem cells through flow cytometry. The other optimization method is to finely adjust a differentiation scheme and increase the yield of A9 dopaminergic neurons; this is a method that can fundamentally solve the heterogeneity problem. To complete this process, the precise regulation of multiple factors with respect to time and space is required.

Additionally, the use of MSC-EVs can also be a novel and efficient therapy. However, the potential mechanisms through which MSC-EVs mediate beneficial effects are unclear, and rigorous studies have demonstrated that the effects of EVs derived from various stem cells or the administration of EVs at various stages of a disease are different. Therefore, thorough research should be conducted to study the effects of derivation or administration stage on the effects of EVs. Furthermore, advances in the EV field are likely to lead to the development of novel administration methods (e.g., nasal sprays containing therapeutic EVs) for curing neurodegenerative diseases in the future, and such methods can deliver the therapeutic cargo of EVs to neurons and glia to promote neurogenesis or modulate neuroinflammation.

The studies that have mainly focused on improving the quality of intrinsic donor cells have not been able to guarantee the effects of stem cell therapy. A point that is overlooked is that the immunogenic and inflammatory reactions induced by PD itself or the injuries that occur during cell transplantation can make a host brain hostile to grafted cells after transplantation, which hampers the appropriate survival, differentiation, maturation, and function of the grafted cells. Grafted cells are mostly transplanted into a proinflammatory microenvironment, and the transplantation process may induce a proinflammatory response or even result in immune rejection. During this process, cytokines may play different functions in different environments or stages. Therefore, the possibility of exploiting the beneficial effects of cytokines and neutralizing those cytokines with a negative effect must be explored. However, several intractable problems exist. First, the safe dose levels for avoiding side effects should be clarified because the overexpression of specific endogenous molecules may influence a normal brain environment. Crucially, an appropriate balance of modulatory factors must be maintained. To achieve this, the mechanisms that control the inhibition versus stimulation of neurogenesis during neuroinflammation should be carefully and comprehensively studied. The identification of the relevant signals provides an opportunity for grafted cells to replicate the process of neurogenesis in vivo.

The epigenetic changes that may influence stem cell transplantation should also be considered. Genetic and epigenetic changes that occur when stem cells are cultured in vitro and passaged prior to transplantation can affect the therapeutic efﬁciency of these cells following transplantation. The loss of expression of specific genes during in vitro cell culturing causes problems relating to whether and when transplanted stem cells can differentiate into dopaminergic neurons in vivo [51]. For example, in a study, iPSCs were transplanted into the putamen of MPTP-treated PD monkeys, which were then divided into two groups on the basis of whether they had excellent or poor TH+ cell innervation; the PD monkeys that had excellent innervation exhibited the upregulation of 11 speciﬁc genes, among which the most prominent was Dlk1, a gene that has a role in facilitating the migration of dopaminergic neurons in the midbrain. That study demonstrated the key role of the epigenetic changes that occur in stem cell transplantation in the upregulation of genes related to dopaminergic neuron differentiation [52]. In another study, transplanted hESCs were predifferentiated for 16, 20, and 23 days into the striatum of rats; notably, the cells that were allowed to predifferentiate for 16 days prior to grafting exhibited tumor growth in vivo, but those that were predifferentiated for 20 days or more did not exhibit this adverse effect; this finding verified the crucial role of the differentiation state of grafted cells and the need to recognize epigenetic markers that indicate a propensity for cancer development or the ability to differentiate into dopaminergic neurons [53]. The epigenetic state of stem cells or the dopaminergic neuronal lineage of the cells gained through the differentiation of stem cells before transplantation into the PD brain may be inconsistent, and this affects treatment outcomes. This may explain why stem cell therapies for PD exhibit varying levels of efﬁcacy. Their results may be affected by unknown epigenetic factors that influence the progression of PD or the viability of transplanted cells, and researchers should thus focus more on stem cell epigenetics to clarify the mechanisms by which epigenetic changes affect stem cell transplantation.

In addition to the aforementioned findings, more advanced tools and techniques are required to deepen our understanding of stem cell differentiation. The developmental trajectory of stem cells on temporal and spatial scales must be examined. The key questions that should be explored include the timing of cell migration, the type of cells that migrate, the sites that cells migrate from and to, what cells transform into when they reach their destinations, and the functions they have at these destinations. The combination of numerous techniques (e.g., spatially resolved transcriptomics and cell lineage tracing) may be helpful. During the differentiation of cells, the molecules that define cell fate and the crosstalk between these signaling pathways are crucial for controlling stem cell differentiation. The construction of a network for analyzing the functions of and the associations between the aforementioned signaling molecules is a meaningful objective. In this regard, techniques such as genome-wide association studies may be helpful. In addition, epigenetics has recently attracted the attention of researchers. Numerous molecules are regulated by specific noncoding RNAs, and thus, clarifying the associations among the chromatin state, transcription factors, and signaling molecules is a useful strategy. Moreover, a brain microenvironment is influenced by multiple factors. Glia cells, which populate numerous cells in the CNS, may play a key role in regulating neuroinflammation. They can provide energy, release anti-inflammatory factors, and reduce oxidative stress. Therefore, we should identify the crosstalk and association between neurons and glial cells and maintain appropriate proportions of various cells during differentiation. A core task that should be performed is the identification of markers that can recognize and define the activated or inhibited state of glial cells.

For clinical research, the topics to address include the identification of the optimal cell type and purity and the exploration of immunosuppression and cell delivery methods. With an increasing understanding of stem cell differentiation, neuroinflammation, and other related knowledge, stem cell therapy may become the main strategy for treating PD in the near future.
